# Simulation of Sound Waves Using the Lattice Boltzmann Method for Fluid Flow: Benchmark Cases for Outdoor Sound Propagation

**DOI:** 10.1371/journal.pone.0147206

**Published:** 2016-01-20

**Authors:** Erik M. Salomons, Walter J. A. Lohman, Han Zhou

**Affiliations:** TNO Netherlands Organization of Applied Scientific Research, Delft, The Netherlands; Newcastle University, UNITED KINGDOM

## Abstract

Propagation of sound waves in air can be considered as a special case of fluid dynamics. Consequently, the lattice Boltzmann method (LBM) for fluid flow can be used for simulating sound propagation. In this article application of the LBM to sound propagation is illustrated for various cases: free-field propagation, propagation over porous and non-porous ground, propagation over a noise barrier, and propagation in an atmosphere with wind. LBM results are compared with solutions of the equations of acoustics. It is found that the LBM works well for sound waves, but dissipation of sound waves with the LBM is generally much larger than real dissipation of sound waves in air. To circumvent this problem it is proposed here to use the LBM for assessing the *excess* sound level, i.e. the difference between the sound level and the free-field sound level. The effect of dissipation on the excess sound level is much smaller than the effect on the sound level, so the LBM can be used to estimate the excess sound level for a non-dissipative atmosphere, which is a useful quantity in atmospheric acoustics. To reduce dissipation in an LBM simulation two approaches are considered: i) reduction of the kinematic viscosity and ii) reduction of the lattice spacing.

## Introduction

In the scientific field of computational fluid dynamics, various numerical methods have been developed for simulating fluid flow. Conventional methods are based on the differential equations for mass and momentum conservation in a fluid, i.e. the continuity equation and the Navier Stokes equations [1,2]. An alternative method is the lattice Boltzmann method (LBM) for simulating fluid flow. The LBM has some advantages over conventional methods of computational fluid dynamics: i) application of the LBM to complex geometries is easy and ii) LBM algorithms are suitable for implementation on parallel platforms. An introduction to the LBM can be found in Ref. [3], which also gives other references to the LBM literature. The basis of the LBM is the Boltzmann equation from kinetic gas theory [1]. With some limiting conditions, LBM solutions agree with solutions of the Navier Stokes and continuity equations [4].

A sound wave traveling in the atmosphere is basically a moving pressure perturbation, and therefore a special case of (compressible) fluid dynamics. This implies that sound propagation may be simulated, in principle, with numerical methods developed in the field of computational fluid dynamics, including the LBM. This is an interesting idea, since it would make it possible to simulate sound propagation in an atmosphere with wind in a *single* simulation. This could be an advantage with respect to the usual two-step approach:

1. first the wind field is calculated by a stationary flow simulation,
2. next the sound field is calculated, using the wind field from step i) as a fixed background field.

The wind field can be used as a fixed field in step ii) because wind speeds are small compared with the sound speed. An example of the two-step approach can be found in Ref. [5], for the case of a noise barrier in an atmosphere with wind. Various methods exist for simulating sound wave propagation in an inhomogeneous atmosphere (step ii), either based on a generalized wave equation [6] or based on the linearized Euler equations [7–10]. An application of the LBM to propagation of plane sound waves is described in Ref. [11]. An extensive study of application of the LBM in acoustics has been performed by Viggen [12–16].

In this article application of the LBM to sound propagation is described and illustrated for various benchmark cases for outdoor sound propagation, including cases with a non-porous ground surface, a porous ground surface, a noise barrier, and atmospheric flow. LBM results are compared with solutions of the equations of acoustics. It is found that the LBM works well for sound waves, but dissipation of sound waves with the LBM is generally much larger than real dissipation of sound waves in air. Various dissipation mechanisms play a role in air: viscous and thermal effects, and also effects from vibrational and rotational relaxations of nitrogen and oxygen molecules [6,17]. With the standard LBM, only viscous effects are included. Moreover, the LBM is not stable for small values of the viscosity, so the true dissipation of sound waves in air cannot be reproduced with the LBM [14,15].

To circumvent this problem it is proposed here to use the LBM for assessing the *excess* sound level, i.e. the difference between the sound level and the free-field sound level. The effect of dissipation on the excess sound level is much smaller than the effect on the sound level itself. The excess sound level for a non-dissipative atmosphere is a useful quantity in atmospheric acoustics. A common approach in atmospheric acoustics is to express the sound level *L* at a receiver as follows [6]:

$$L=L_{\text{free}}\;+\Delta L,$$ (1)

where *L*_free_ is the free-field sound level and Δ*L* is the excess sound level. Eq (1) is in fact the definition of the excess sound level. The free-field sound level is the sound level in a unbounded homogeneous atmosphere, where sound waves are attenuated only by geometrical spreading and dissipation in air. The excess attenuation represents effects from the ground, obstacles, and atmospheric refraction. In many practical outdoor situations all sound paths (direct, reflected, and diffracted paths) have approximately equal lengths, and the overall effect of dissipation can be approximated by the free-field dissipation contained in the term *L*_free_. This means that a non-dissipative atmosphere can be assumed for the excess attenuation.

This work was performed within the framework of the European ITEA2 project MACH, which aims at optimizing scientific calculations on various computer platforms [18]. One of the fields of application considered in MACH is computational fluid dynamics. The LBM is explored in MACH because the LBM algorithm is suitable for implementation on parallel computer platforms. Previous work on speeding up LBM models by means of parallel platforms, including the Graphical Processing Unit (GPU) of a personal computer, is described in Refs. [19,20]. The GPU has also been used for speeding up other methods of computational fluid dynamics [21]. The present article is an extended version of a paper presented at the conference ‘Particles 2015’ [22].

## Methods

In this section, elements of the LBM are summarized. First the LBM for fluid flow is briefly described, and the benchmark case of a lid-driven cavity is considered. Next application of the LBM to sound propagation in described.

### LBM for Fluid Flow

A basic quantity of the LBM is the particle distribution function [3]. A regular lattice is used and the values of the distribution function at the nodes of the lattice represent the local density of fluid particles at the nodes. Different particle velocities are distinguished, corresponding to the movement of particles to different neighboring lattice nodes during a time step. Consequently, the particle distribution function varies not only with position on the lattice but is also a function of the discrete set of particle velocities.

**Fig 1:**
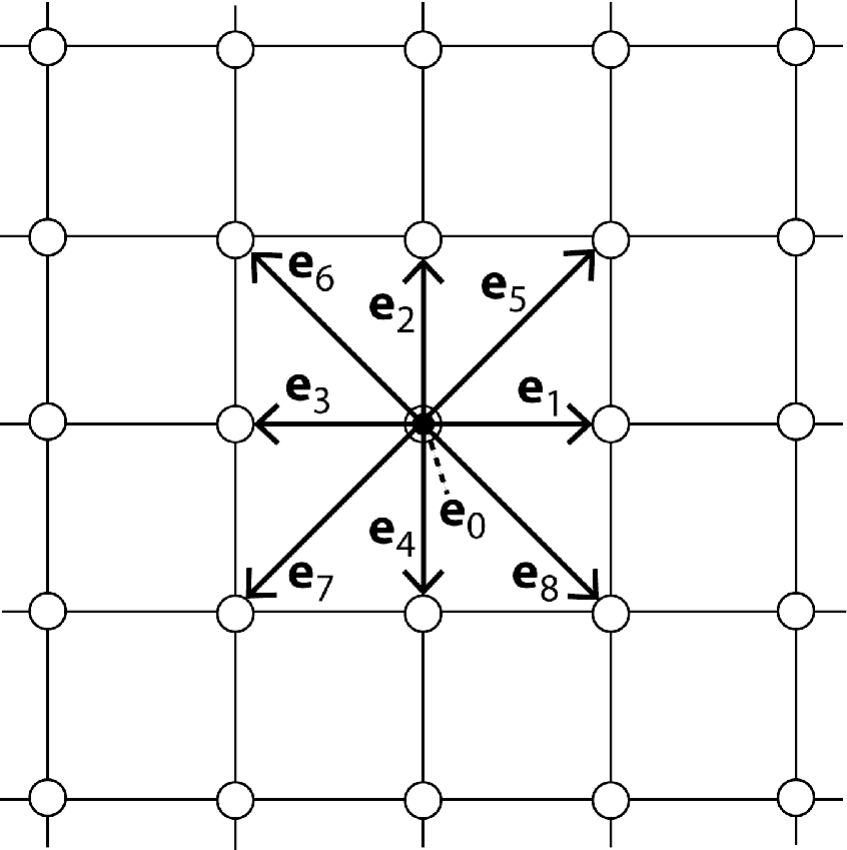
Rectangular 2D lattice illustrating the D2Q9 scheme for the LBM.

Fig 1 illustrates the widely used D2Q9 lattice for the LBM in 2D. There are nine velocity vectors **e**_*i*_ to neighboring lattice nodes: **e**_0_ = (0,0), **e**_1_ = (1,0), **e**_2_ = (0,1), …, **e**_8_ = (1,-1). Here an *xy* coordinate system is used with a horizontal *x* axis and a vertical *y* axis. The first vector **e**_0_ corresponds to zero velocity, so particles with *i* = 0 do not move to a neighboring node. In this article also the D3Q19 lattice for the LBM in 3D is considered, with 19 velocity vectors [23,24].

The LBM particle distribution function is denoted as *f*_*i*_(**x**,*t*), where index *i* distinguishes the velocity vectors, **x** denotes position on the lattice, and *t* represents time. The LBM formulation considered here is based on the BGK (Bhatnagar-Gross-Krook) approximation. The evolution of the fluid is represented by the following equation:

$$f_{i}(x+e_{i}\Delta t,t+\Delta t)=f_{i}(x,t)-\tau^{-1}\left(f_{i}(x,t)-f_{i}^{\text{eq}}(x,t)\right),$$ (2)

where **e**_*i*_ are the velocity vectors to neighboring nodes, Δ*t* is the time step, *τ* is the relaxation time, and *f*_*i*_^eq^ is the local equilibrium distribution function. Length and time units are chosen such that lattice spacing Δ*x* and time step Δ*t* are unity, so the particle speed *c* = Δ*x*/Δ*t* is also unity. The first part of the equation, *f*_*i*_(**x** + **e**_*i*_Δ*t*,*t* + Δ*t*) = *f*_*i*_(**x**,*t*), represents streaming of particles between neighboring nodes. The last term represents collisions between particles. Collisions of the fluid particles correspond to a relaxation towards local equilibrium.

The macroscopic fluid density *ρ* and the fluid velocity **u** follow from the distribution function: *ρ* = ∑_*i*_ *f*_*i*_ and **u** = *ρ*^−1^∑_*i*_ *f*_*i*_**e**_*i*_. The fluid density is related to the pressure *p* through the ideal gas law (at constant temperature) $p=c_{s}^{2}\rho$, where *c*_*s*_ is the sound speed in lattice units Δ*x*/Δ*t*, which is 1/√3 for the D2Q9 and D3Q19 lattices. The density is included in the LBM as an independent variable in a regime close to fluid incompressibility [4]. The relaxation time *τ* is related to the kinematic shear viscosity *ν* of the fluid: $\nu =c_{s}^{2}\left(\tau -\frac{1}{2}\right)$. The viscosity is positive for $\tau >\frac{1}{2}$. As *τ* approaches $\frac{1}{2}$ numerical difficulties may occur. A safe choice is *τ* = 1, which gives $\nu =\frac{1}{6}$. In the BGK approximation, the equilibrium distribution function is given by

$$f_{i}^{\text{eq}}=w_{i}\rho \left(1+\frac{e_{i}\cdot u}{c_{s}^{2}}+\frac{{\left(e_{i}\cdot u\right)}^{2}}{2c_{s}^{4}}-\frac{u^{2}}{2c_{s}^{2}}\right),$$ (3)

which is a truncated expansion of the Maxwell velocity distribution. The factors *w*_*i*_ are weight factors for the different velocity directions. For the D2Q9 scheme, the weights *w*_*i*_ are: 4/9 for *i* = 0, 1/9 for *i* = 1-4, and 1/36 for *i* = 5-8.

The core of an LBM algorithm is a loop over four elements:

- collisions
- streaming
- boundary conditions
- macroscopic quantities.

The first three elements yield updates of the distribution function, due to collisions, streaming, and boundary conditions, respectively. The fourth element is the calculation of density *ρ* and fluid velocity **u**, which are used in the collision calculation.

Different types of boundary conditions exist. At a stationary boundary (zero velocity at a no-slip wall) one may apply bounceback conditions, which means that a distribution function component *f*_*i*_ directed into the fluid is obtained from the component in the opposite (outward) direction. First-order and second-order bounceback formulations have been developed [3,24]. For the calculations presented in this article, first-order bounceback conditions were applied, with the boundary lattice nodes located exactly at the solid boundary. For example, at a north boundary of a D2Q9 system, components *f*_4_, *f*_7_, and *f*_8_ are calculated as follows: *f*_4_ = *f*_2_, *f*_7_ = *f*_5_, and *f*_8_ = *f*_6_. For a boundary with an imposed velocity or pressure, boundary conditions are presented in Ref. [25]; see also Refs. [23,24]. For the calculations presented below, a horizontal velocity was imposed at the north boundary of a D2Q9 system with the following boundary conditions: *f*_4_ = *f*_2_, $f_{7}=f_{5}+\frac{1}{2}(f_{1}-f_{3})-\frac{1}{2}\rho_{\text{N}}u_{0}$, and $f_{8}=f_{6}+\frac{1}{2}(f_{3}-f_{1})+\frac{1}{2}\rho_{\text{N}}u_{0}$, where *u*_0_ is the imposed velocity and *ρ*_N_ = *f*_0_ + *f*_1_ + *f*_3_ + 2(*f*_2_ + *f*_5_ + *f*_6_) is the density at the boundary.

**Fig 2:**
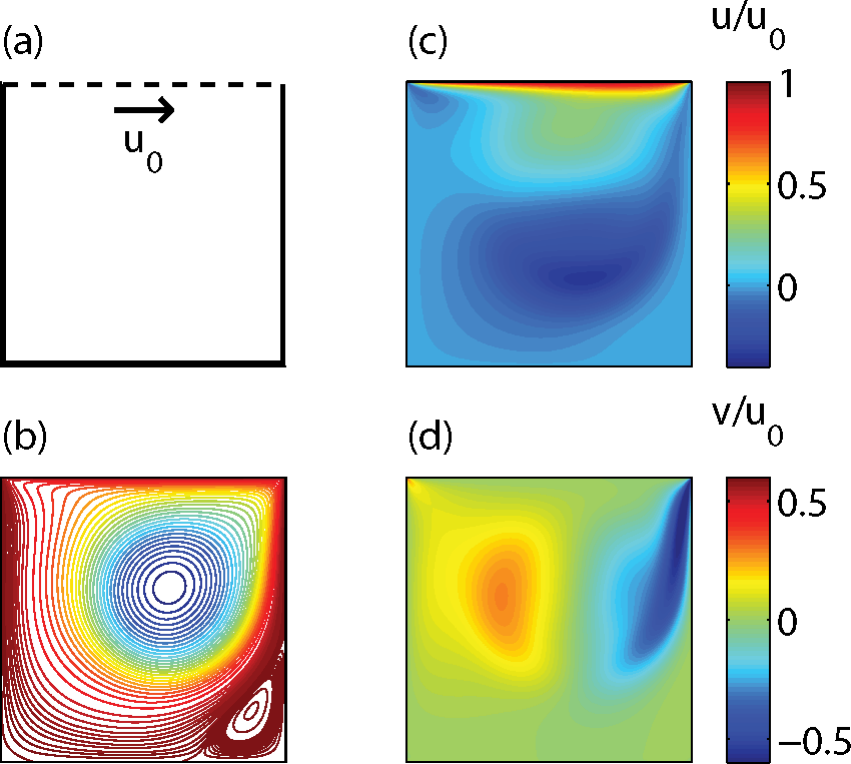
Results of an LBM calculation for a lid-driven cavity, with the geometry (a), streamlines (b), and normalized velocity components *u*/*u*_0_ (c) and *v*/*u*_0_ (d).

Fig 2 shows results of a D2Q9 LBM calculation for the case of a lid-driven cavity in 2D. The fluid is enclosed in a square box with solid walls, while at the top of the box a constant horizontal velocity *u*_0_ is imposed as a boundary condition. At the solid walls first-order bounceback conditions were applied. At the top the velocity boundary condition described above was used. The calculation was performed for a 1000x1000 lattice, with *u*_0_ = 0.06 and *ν* = 0.06. The Reynolds number Re = *u*_0_*L*_lat_/*ν* is equal to 1000 in this case, with lattice size *L*_lat_ = 1000. The graphs show the (steady state) fluid flow after 16 ⋅ 10^4^ time steps.

### Calculation Times

This section presents LBM calculation times and speeds for 2D and 3D cases of a lid-driven cavity. First calculation times and speeds are given for non-optimized codes and next calculation times and speeds are given for optimized codes. Calculation speed is expressed in units of MLUPS (mega lattice site updates per second). For example, if 1 second is required for one update of a lattice with 10^6^ lattice sites, the speed is 1 MLUPS.

Non-optimized codes were developed in Matlab, C/C++, Fortran, and CUDA, for the D2Q9 case of the lid-driven cavity. The codes were run on a PC with a 2.4 GHz CPU (Intel Xeon E5620), 6 GB RAM, and an NVIDIA GPU (GeForce GTX 680, 4 GB, 1536 CUDA cores).

For a 100x100 lattice with Re = 1000 (*u*_0_ = 0.6, *ν* = 0.06), a steady state was reached within 4000 time steps. This took about 20 seconds with the Matlab code, 8 seconds with the C/C++ code, and 6 seconds with the Fortran code (codes were run on a single CPU core). These times correspond to calculation speeds of 2, 5, and 7 MLUPS, respectively. With the CUDA code running on the GPU a calculation time of 2 seconds was found, corresponding to 20 MLUPS.

For a D2Q9 1000x1000 lattice with Re = 1000 (*u*_0_ = 0.06, *ν* = 0.06) 16 ^**.**^ 10^4^ time steps were needed to reach a steady state, and with a Matlab code this took 32 hours (on a single CPU core), corresponding to 1.4 MLUPS. With a modified Matlab code that employs the GPU, making use of the Matlab parallel processing toolbox, the time was 12 hours, corresponding to 3.7 MLUPS. The total number of 16 ^**.**^ 10^4^ time steps is almost two orders of magnitude larger than the 4000 times steps for the 100x100 lattice, as the equilibration time scales with the square of the linear lattice size [26].

For a D3Q19 100x100x100 lattice with Re = 1000 (*u*_0_ = 0.6, *ν* = 0.06) a steady state was reached within 4000 time steps, which took about 6000 seconds with a Matlab code (on a single CPU core), corresponding to 0.7 MLUPS. With Matlab running on the GPU the calculation time was about 1800 seconds, corresponding to 2.2 MLUPS.

Finally, optimized CUDA codes for the lid-driven cavity were developed and run on a PC with a 2.4 GHz CPU (Intel Xeon E5-2630 v3), 48 GB RAM, and an NVIDIA GPU (Tesla K40M, 12 GB, 2880 CUDA cores). The optimization of the CUDA codes was performed along the lines described in Ref. [19] and takes advantage of the GPU architecture. For a D2Q9 96x96 lattice a speed of 410 MLUPS was obtained. For a D3Q19 96x96x96 lattice a speed of 560 MLUPS was obtained (a lattice size of 96 is chosen to match the GPU architecture).

The above values of the calculation time and calculation speed are collected in Table 1. The values illustrate that the LBM calculation speed may be enhanced by two orders of magnitude by parallel computation on the GPU. The reference value for non-parallel computation is between 0.7 and 7 MLUPS in this case, while the calculation speed for parallel computation on the GPU is around 500 MLUPS. It should be noted that the non-parallel codes cannot be optimized much. In particular the Fortran speed of 7 MLUPS cannot be enhanced with the single CPU used here. The Matlab calculation speed is higher for D2Q9 than for D3Q19, due to the fact that the number of velocity vectors per site is smaller for D2Q9 than for D3Q19. The CUDA calculation speed, however, shows the reverse behavior, which illustrates that the optimization of CUDA codes for the GPU is rather complex. The origin of the reverse behavior is that the efficiency of running many GPU calculation threads in parallel is higher for the 96x96x96 D3Q19 system than for the 96x96 D2Q9 system. Further work on the optimization is in progress, within the framework of the European project MACH [18].

**Table 1:** Calculation times and calculation speeds for various LBM calculations.

lattice	time steps	code	calculation time	calculation speed (MLUPS)
D2Q9 100x100	4000	Matlab	20 s	2
D2Q9 100x100	4000	C/C++	8 s	5
D2Q9 100x100	4000	Fortran	6 s	7
D2Q9 96x96	4000	optimized CUDA	0.09 s	410
D2Q9 1000x1000	16 ^**.**^ 10^4^	Matlab	32 h	1.4
D2Q9 1000x1000	16 ^**.**^ 10^4^	Matlab parallel	12 h	3.7
D3Q19 100x100x100	4000	Matlab	6000 s	0.7
D3Q19 100x100x100	4000	Matlab parallel	1800 s	2.2
D3Q19 96x96x96	4000	optimized CUDA	6.3 s	560

### LBM for Acoustics

**Fig 3:**
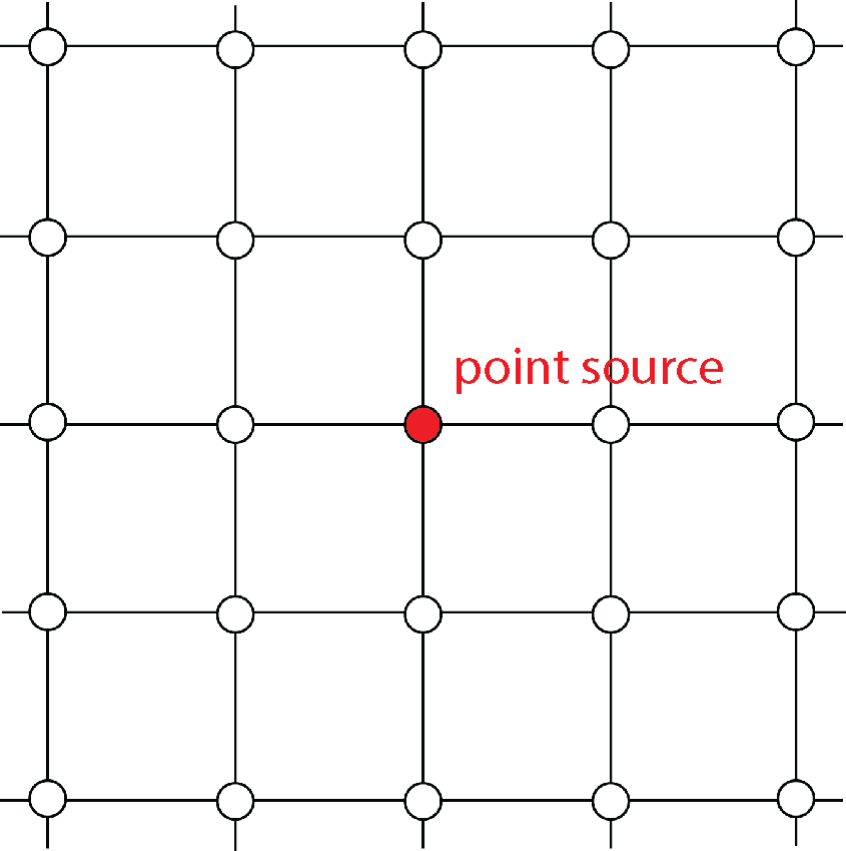
Sound waves are generated by imposing the fluid density at a lattice node, according to a harmonically oscillating function around an equilibrium value.

This section describes how the LBM was used in this study for simulating propagation of sound waves. The D2Q9 LBM model was used to model circular sound waves generated by a point source, located at a single node of the LBM lattice (see Fig 3). Circular waves in 2D correspond to cylindrical waves in 3D.

The fluid density at the source position is imposed at the end of each LBM time step, according to the harmonic function *ρ* = 1 + *B* sin(*ωt*), where *t* is the time, *B* is the amplitude, *ω* = 2*π*/*T* is the angular frequency, and *T* is the harmonic period. For the simulations presented in this article, the values *B* = 0.01 and *T* = 40 were used (in LBM units) unless indicated otherwise. The density oscillates around the equilibrium value of unity. The off-equilibrium density *ρ*' ≡ *ρ* − 1 is called acoustic density. It should be noted that no additional constraints were imposed on the distribution function and fluid velocity at the source position, as the above harmonic constraint on the density was found to result in circular sound waves emanating from a point source. More advanced methods for generating the field of a monopole point source have been described in Ref. [16].

The analytic solution for the (free field) acoustic density is given by (the real part) of the following function of time *t* and distance *r* to the source:

$$\rho '=AH_{0}^{\left(2\right)}\;(kr)\;\text{exp}(i\omega \;t),$$ (4)

where *A* is a constant, *H*_0_^(2)^ is the Hankel function of order zero and second kind, and *k* is the wavenumber. This expression is a stationary solution of the lossy wave equation, taking into account effects of viscosity [12,27]. The wave number is given by *k* = *ω*/*c*_*s*_ - *iα*_*s*_, with $\alpha_{s}=(\omega /\sqrt{2}c_{s})\sqrt{\left[\sqrt{1+{\left(\omega \tau_{s}\right)}^{2}}-1]/[1+{\left(\omega \tau_{s}\right)}^{2}\right]}$. Quantity *τ*_*s*_ is given by $\tau_{s}=c_{s}^{-2}(\frac{4}{3}\nu +\nu ')$, with kinematic shear and bulk viscosities *ν* and $\nu '=\frac{2}{3}\nu$ [28]. For the cases considered here the relation *ωτ*_*s*_ << 1 holds, and the expression for *α*_*s*_ reduces to $\alpha_{s}=\frac{1}{2}\omega^{2}\tau_{s}/c_{s}$. It has been shown theoretically [13] that the LBM yields expressions for the absorption and dispersion of sound waves that agree up to first order in *ωτ*_*s*_ with the expressions derived from the lossy wave equation.

The time and space resolutions Δ*x* and Δ*t* relate quantities in lattice units and physical units.

Constraints are imposed by the physical values of the sound speed *c* and the kinematic viscosity *ν* of the gas that is being simulated, air in this case:

$$\begin{array}{c} c_{\text{ph}}=c_{\text{LBM}}\;\Delta x/\Delta t\\ \nu_{\text{ph}}=\nu_{\text{LBM}}\;\Delta x^{2}/\Delta t \end{array}$$ (5)

where subscript ‘ph’ refers to the physical system and subscript ‘LBM’ refers to the LBM system. Sound speed *c*_ph_ is equal to 340 m/s and sound speed *c*_LBM_ is equal to *c*_*s*_ = 1/√3. If a lattice spacing of Δ*x* = 0.1 m is chosen, the time step is Δ*t* = 1.7 ^**.**^ 10^−4^ s. This implies that the frequency *f* = 1/*T* of the sound wave is equal to 147.2 Hz (since *T* = 40 in LBM units). For *ν*_LBM_ a value of 0.06 will be used (unless indicated otherwise), which yields a value of 3.5 m^2^/s for *ν*_ph_. The real value of *ν*_ph_ for air is much smaller: 1.6 ^**.**^ 10^−5^ m^2^/s. As will be shown in a later section, the value of *ν*_LBM_ can be reduced a bit, but not down to values that would be realistic for air.

It should be noted that the multiple relaxation time (MRT) formulation of the LBM allows lower values of the kinematic viscosity than with the BGK formulation used here [29,13]. Numerical results presented in Ref. [29] show that the MRT LBM is stable down to four times lower values of the kinematic viscosity. In Ref. [13] it is shown that for sound propagation the MRT LBM performs better than the BGK LBM does.

## Results and Discussion

### Free Field

**Fig 4:**
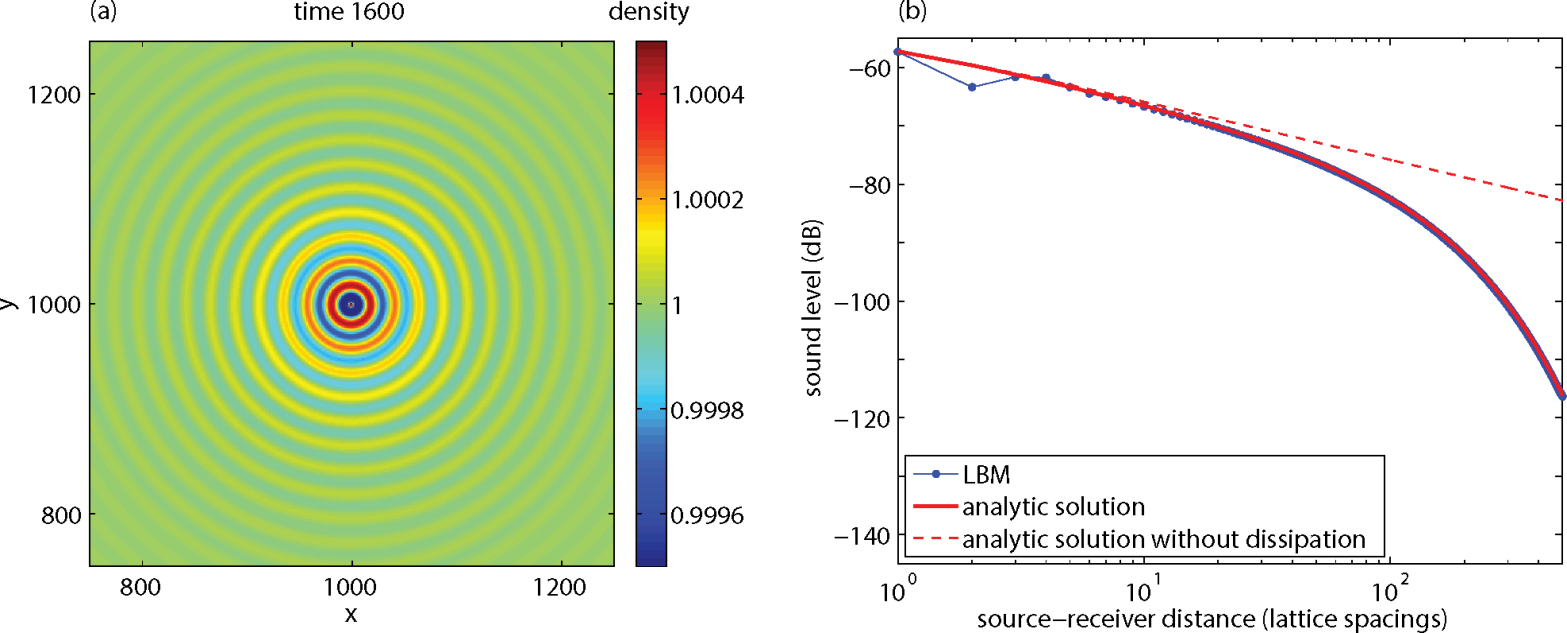
Results of an LBM calculation for a sound wave, showing the sound field at time 1600 (a) and the sound level as a function of source-receiver distance (b).

Fig 4A shows the sound field at time step 1600, generated by a point source at position (1000,1000) in a 2000x2000 lattice with *ν* = 0.06. The wavelength is equal to *T* ⋅*c*_*s*_, which is 23 lattice units in this case with *T* = 40, in agreement with the wave pattern shown in Fig 4A. The amplitude of the wave decreases with distance *r* from the source by two effects, geometrical spreading and dissipation. Geometrical spreading is represented by the factor *r* ^-1/2^, which is the asymptotic behavior of the Hankel function in Eq (4) for real wavenumber *k*. Dissipation due to viscosity is represented by the imaginary part *α*_*s*_ of the wave number.

The graph in Fig 4B shows how the sound level *L* decreases with distance *r*. Here the sound level is defined as *L* = 20 lg *ρ*'_rms_, with *ρ*'_rms_ the rms-value of *ρ*'. It should be noted that the absolute values of *L* are not of interest, so the reference value of *ρ*' in the definition of the absolute sound level [6] has been omitted here for simplicity. The LBM results agree with the analytic solution (4) beyond a distance of three lattice spacings (blue dots and the red solid line coincide here). Also shown is the analytic solution without dissipation (*α*_*s*_ = 0), which decays with 10 dB per distance decade (20 lg 10^−1/2^ = −10 dB). The graph demonstrates that dissipation dominates the attenuation at large distance, where the attenuation reaches a value of about 0.07 dB per lattice spacing in this case.

The dissipation is a problem for direct application of the LBM in acoustics. The dissipation of sound waves in air is small, typically 10^-4^ dB/m at frequency 50 Hz, 10^-3^ dB/m at 250 Hz, and 10^-2^ dB/m at 2 kHz. For practical situations with propagation distances less than 100 m, the dissipation attenuation is therefore less than 1 dB, which can often be neglected. The dissipation attenuation in the LBM system is much larger. If a lattice spacing of 0.1 m is chosen, for example, the above value of 0.07 dB per lattice spacing corresponds to 0.7 dB/m at frequency 147.2 Hz. Consequently, the main challenge here is to reduce the dissipation attenuation in the LBM system.

There are basically two ways to reduce the dissipation attenuation in the LBM system:

1. reducing the lattice spacing Δ*x*, i.e. increasing the number of lattice points for a given system size in meters,
2. reducing the kinematic viscosity *ν*.

This is explained below.

To explain the effect of reducing the lattice spacing Δ*x*, the values Δ*x* = 0.1 m, Δ*t* = 1.7 ^.^ 10^−4^ s, *f* = 147.2 Hz, and *T* = 40 are considered (see previous section). If the lattice spacing Δ*x* is halved, then the time step Δ*t* is also halved, and therefore harmonic period *T* must be doubled to obtain the same frequency of 147.2 Hz. Consequently, *ω* = 2*π*/*T* is halved and *α*_*s*_ is reduced by a factor of 4, as follows from the relation $\alpha_{s}=\frac{1}{2}\omega^{2}\tau_{s}/c_{s}$. This implies that the attenuation per lattice spacing is reduced by a factor of 4, so the attenuation per meter is reduced by a factor of 2.

The effect of reducing the kinematic viscosity *ν* is more straightforward: *α*_*s*_ is proportional to *τ*_*s*_, and therefore proportional to *ν*. As indicated before, the LBM is unstable for small values of *ν*, typically below *ν* = 0.01.

It is concluded here that direct application of the LBM in acoustics is restricted to cases with small propagation distances and low sound frequencies (dissipation parameter *α*_*s*_ is proportional to the squared frequency). The LBM application range, i.e. the range of distances and frequencies where dissipation effects are small, depends on the chosen lattice spacing or number of lattice points. The LBM application range can be enhanced by working with the excess level, as illustrated in the following sections.

### Effect of a Non-Porous Ground Surface

**Fig 5:**
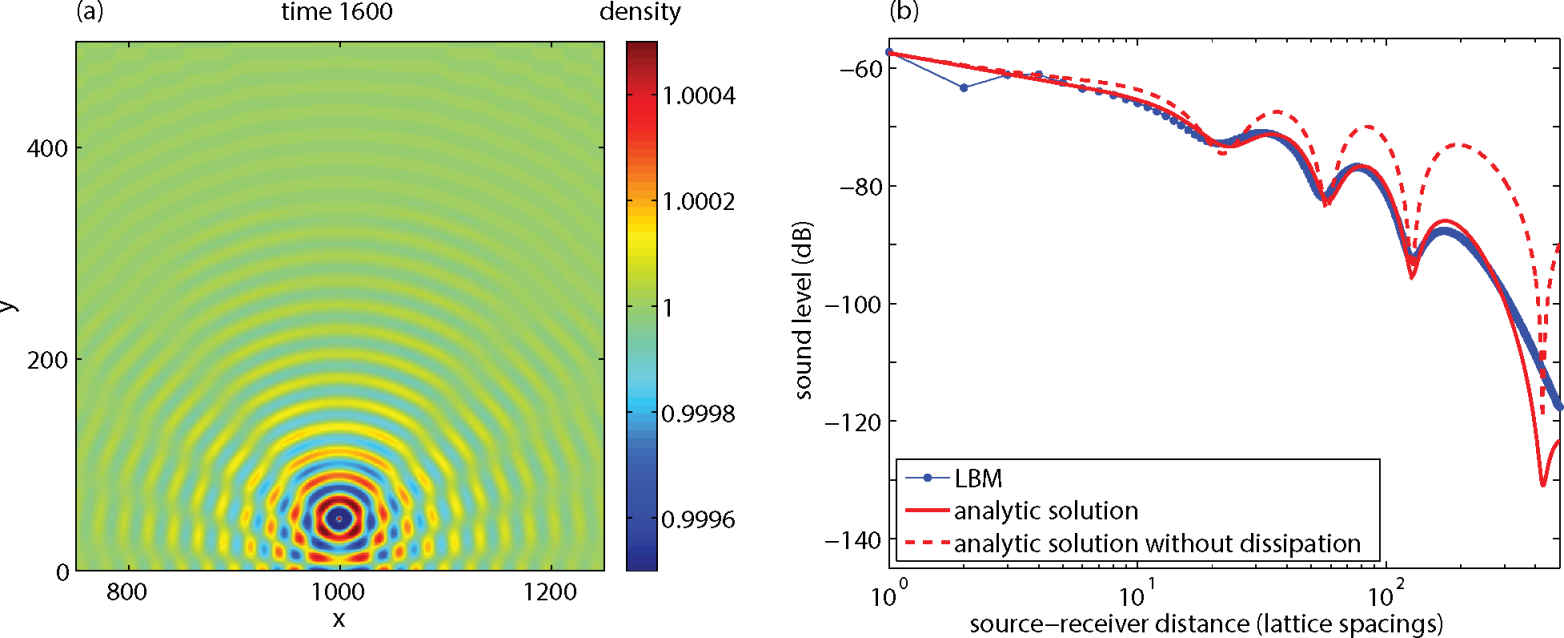
As Fig 4, for a point source and receivers at height *y* = 50.

Fig 5 shows the results of a simulation of the sound field generated by a point source near a non-porous ground surface. The system is similar to the previous system, except now a 2000x500 lattice was used and the source was located at position (1000,50), so the source is 50 lattice spacings above the ground surface. The ground surface is treated as a solid wall, where first-order bounceback conditions were applied. The effect of interference between direct and reflected sound waves is visible as spatial oscillations of the sound level in the graph in Fig 5B. The sound level was sampled at receivers at positions (1001–1500,50).

**Fig 6:**
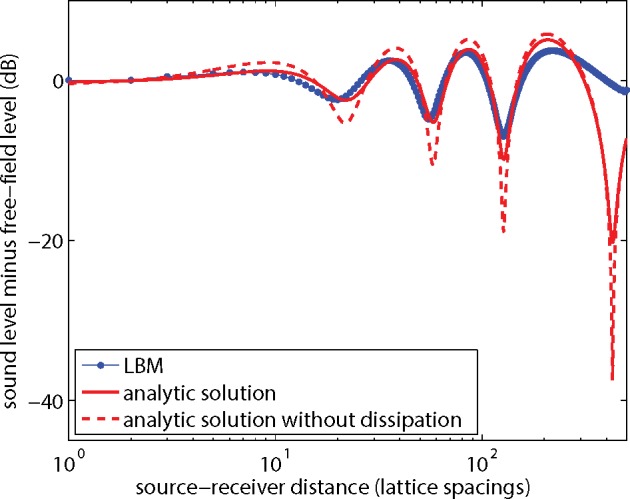
LBM results and analytic solutions from Fig 5B, expressed as the excess sound level Δ*L* (sound level minus free-field level) as a function of distance.

The analytic solutions in Fig 5 were calculated by (coherent) summation of the direct field and the field reflected by the ground surface. The LBM results agree well with the analytic solution (with dissipation), although there are some deviations. The results from Fig 5B are also represented in Fig 6, now expressed as the excess sound level Δ*L*, defined as the sound level minus the free-field sound level. The dissipation effects largely cancel with this level difference. As described in the introduction of this article, it is common practice in acoustics to work with excess level Δ*L* for an atmosphere without dissipation.

In a following section it will be shown how the remaining dissipation effects in Fig 6 can be further reduced, and also how the deviation of the LBM results from the analytic solution in Fig 6 can be further reduced.

It is of interest to note that the difference in Δ*L* between 2D solutions and 3D solutions is in general small [7]. This was confirmed here by recalculating the analytic solutions in Fig 6 with exp(−*ikr*)/*r* instead of $H_{0}^{\left(2\right)}(kr)$, since exp(−*ikr*)/*r* is the 3D analogue of the 2D solution $H_{0}^{\left(2\right)}(kr)$.

The deviations between LBM results and the analytic solution in Figs 5 and 6 may originate from the fact that the LBM yields expressions for the absorption and dispersion of sound waves that agree with the expressions derived from the lossy wave equation only up to first order in *ωτ*_*s*_, as indicated before. In addition, viscous boundary layer effects may play a role [17], as the boundary layer thickness is larger than in reality due to the high viscosity used here.

### Effect of a Porous Ground Surface

**Fig 7:**
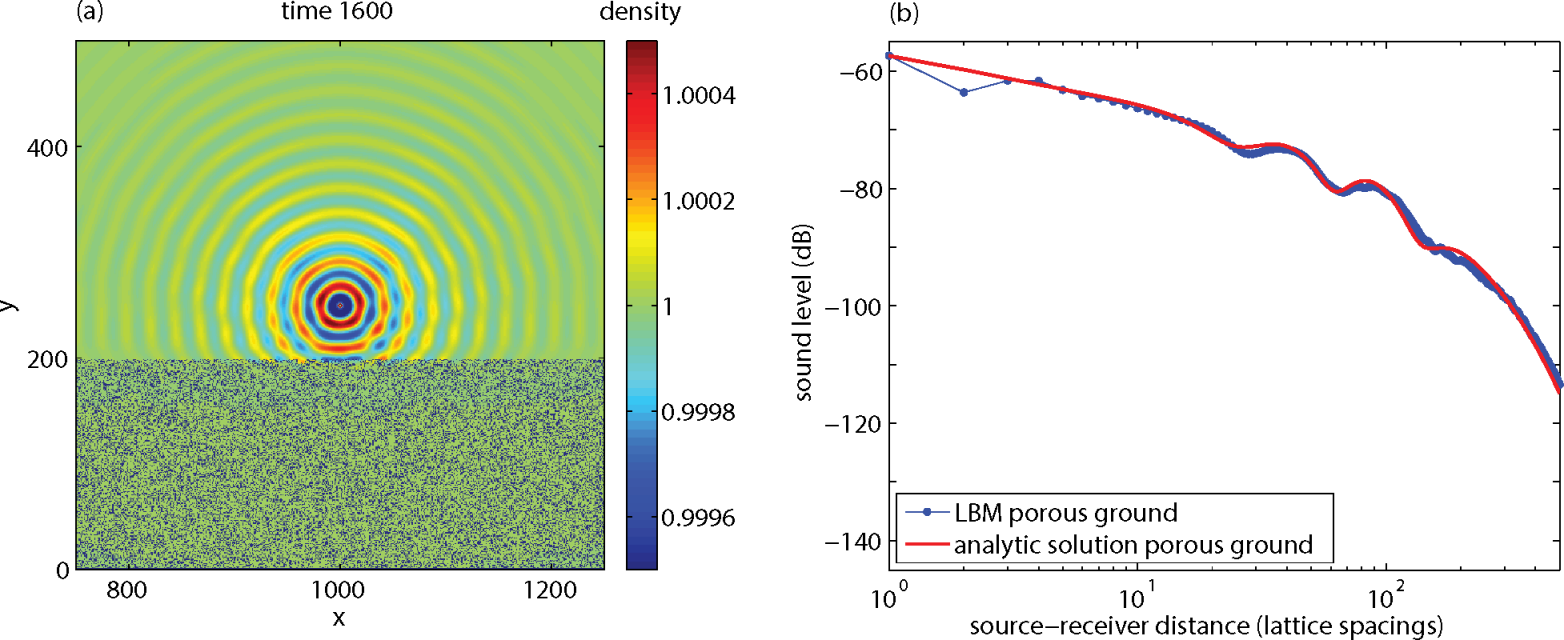
Results of an LBM calculation for a source and receivers at *y* = 250 and a porous medium below *y* = 200, showing the sound field at time 1600 (a) and the sound level as a function of distance (b).

Fig 7 shows the results of a simulation of the sound field generated by a point source near a porous ground surface. A system with a 2000x700 lattice was used, with numerical parameters as before. The region between *y* = 0 and *y* = 200 was filled with a porous medium, and source and receivers were located at *y* = 250. The porous medium was generated by randomly selecting 30 percent of the nodes as solid nodes, where LBM bounceback conditions apply. The interference pattern in Fig 7B is less pronounced than in Fig 5B for the non-porous ground, since the reflection from the porous ground is weaker.

**Fig 8:**
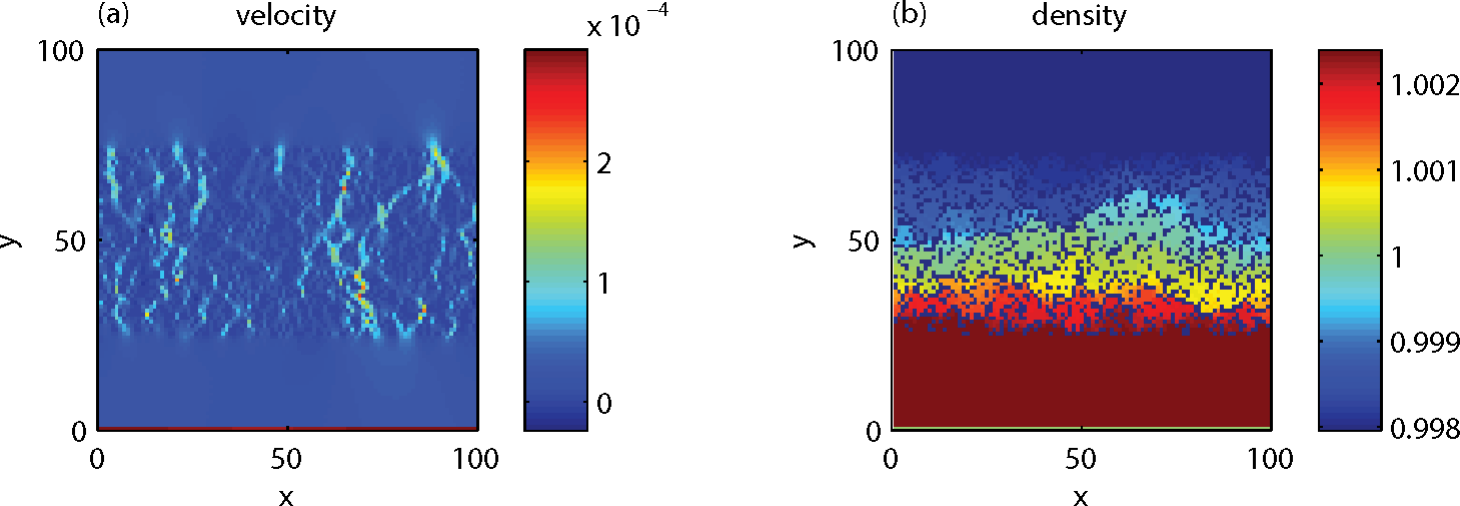
Stationary vertical velocity field (a) and density field (b) in a 100x100 LBM system with a porous medium between *y* = 25 and *y* = 75 and an upward inflow condition at *y* = 0, used for estimating the flow resistivity.

The analytic solution in Fig 7B was calculated with a theoretical model for sound reflection from a porous ground (see Ref. [6]), where again a lattice spacing of 0.1 m was assumed to convert LBM units to physical units. The total sound field is the sum of the direct field and the field reflected by the ground surface. The acoustic impedance of a porous ground is a function of the flow resistivity, the porosity, and a structure factor [30] (see also [6,7]). In this case, the porosity is 0.7, for the structure factor a value of 3 was used, and the flow resistivity was estimated at 10 kPa s m^-2^ from an LBM simulation illustrated in Fig 8. For this simulation a 100x100 lattice was used, with porous medium between *y* = 25 and *y* = 75, and an upward inflow condition at *y* = 0. The inflow condition was achieved by adding 0.001 to the upward velocity at *y* = 0 during each time step, which resulted in a homogeneous velocity field near the porous layer. Periodic boundary conditions were applied at the boundaries of the system. The flow resistivity was estimated from the definition as the ratio of the pressure gradient and the velocity.

**Fig 9:**
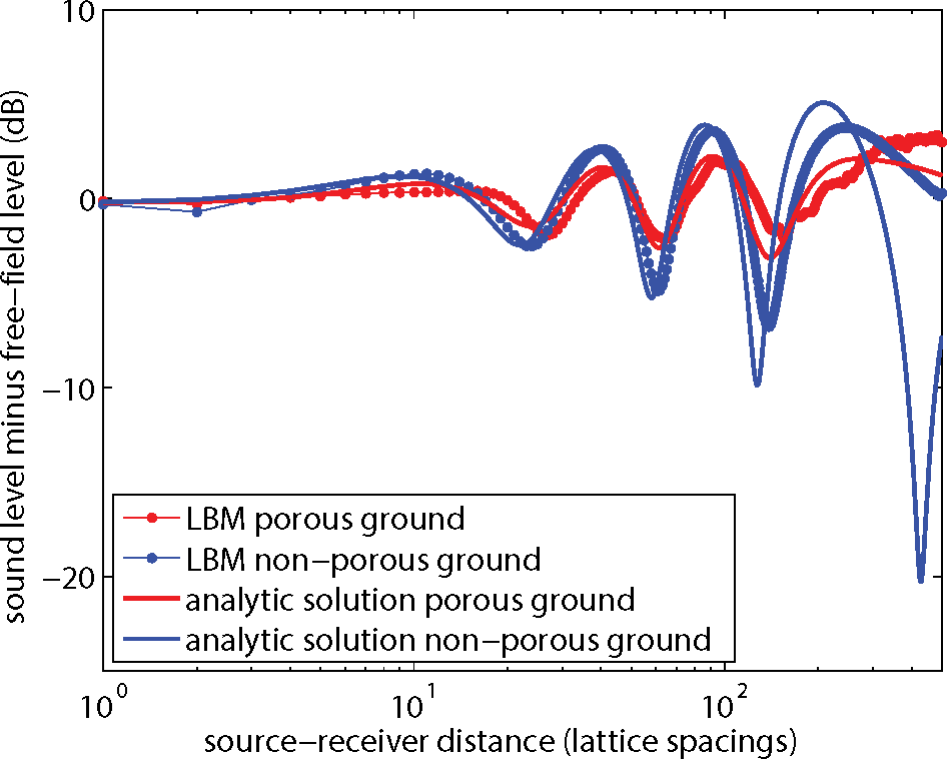
LBM results and analytic solutions for porous and non-porous ground, expressed as the excess sound level Δ*L* as a function of distance.

Fig 9 compares the results for porous and non-porous ground in terms of excess sound level Δ*L*. Both the LBM results and the analytic solutions show that the interference minima and maxima are more pronounced for the non-porous ground than for the porous ground.

### Effect of a Noise Barrier

**Fig 10:**
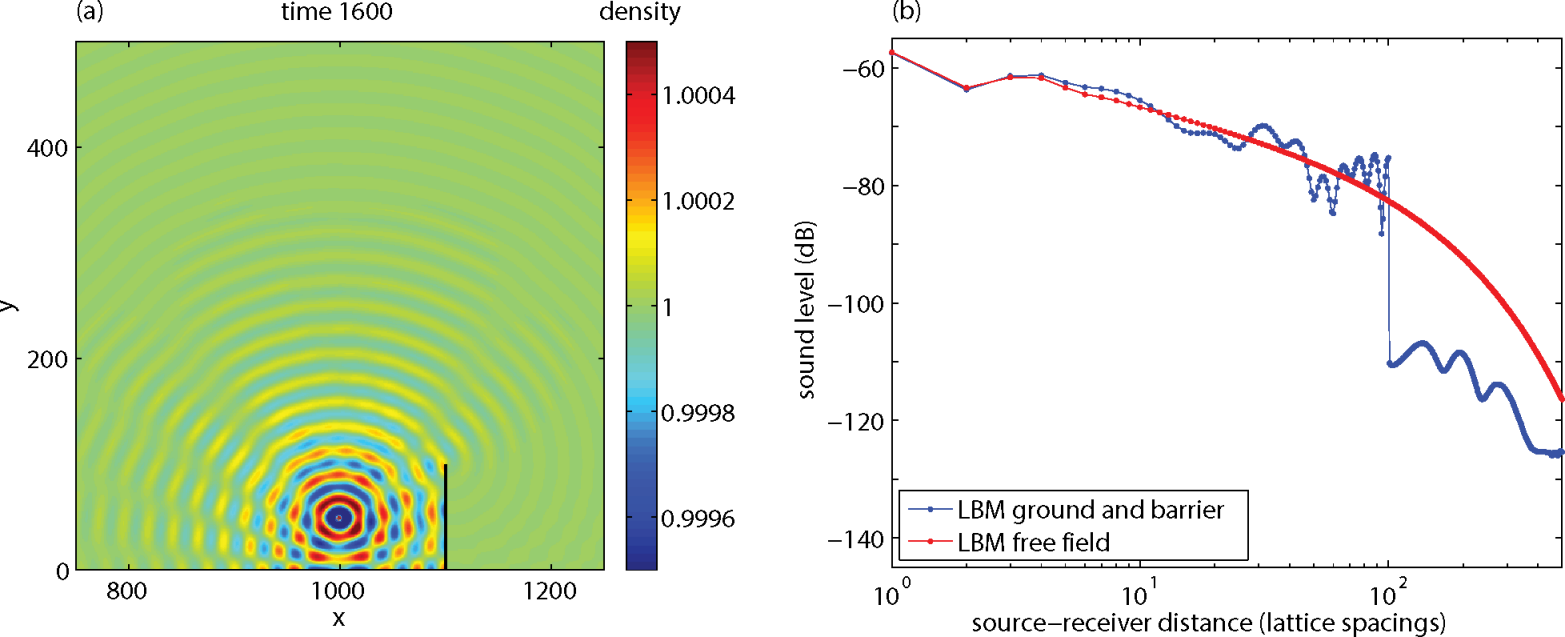
Results of an LBM calculation for a non-porous ground surface with a noise barrier at *x* = 1100 (black line), showing the sound field at time 1600 (a) and the sound level as a function of distance (b).

Fig 10 shows the results of a simulation of the effect of a noise barrier on a non-porous ground surface. A system with a 2000x1000 lattice was used, with numerical parameters as before. The source was located at position (1000,50). The noise barrier was located at position *x* = 1100, with a height of 100 lattice spacings. First-order bounceback conditions were applied at the surface of the noise barrier. Receivers were located at positions *x* = 1001 to *x* = 1500, at a height of *y* = 50. Fig 10B shows that the effect of the noise barrier is a decrease of the sound level by about 30 dB.

**Fig 11:**
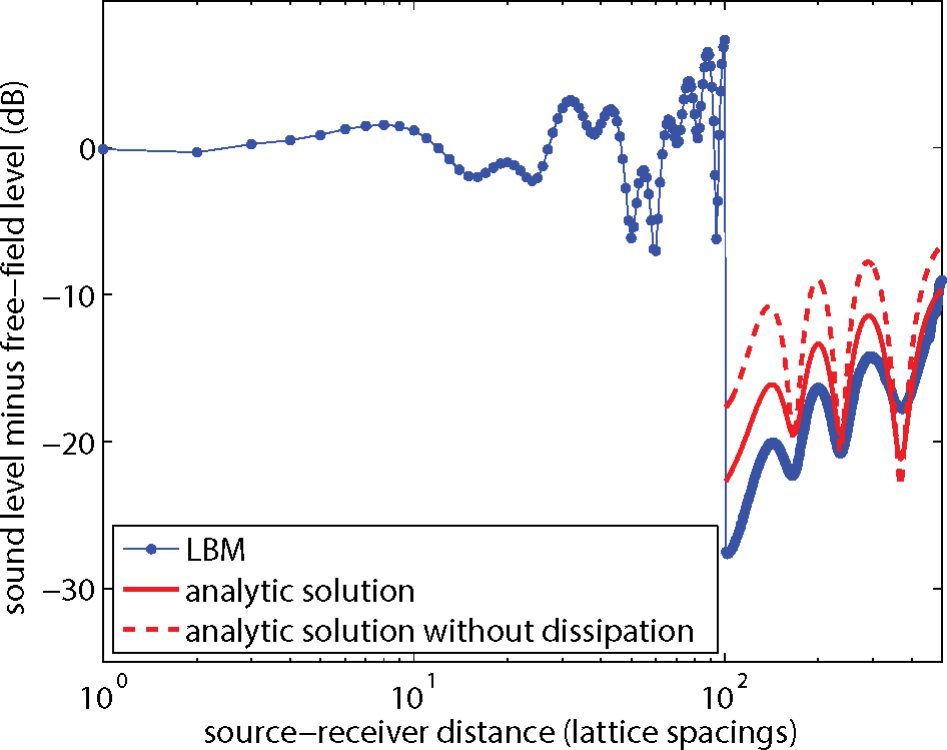
LBM result from Fig 10 and analytic solutions, expressed as the excess sound level Δ*L* as a function of distance.

In Fig 11 the results are shown in terms of the excess sound level Δ*L*. Also included are analytic solutions for receivers behind the barrier, with and without dissipation. The solutions are based on a theory of diffraction of spherical sound waves by wedge-shaped objects [31,32]. The LBM results deviate a bit from the analytic solution (with dissipation) and also the two analytic solutions deviate from each other. Again, viscous boundary layer effects may have affected the LBM results. In the next section it will be shown how the remaining dissipation effects, and also the deviation of the LBM results, in Fig 11 can be further reduced.

### Dissipation

The results in the previous sections show that the effect of dissipation can be reduced by working with the excess sound level Δ*L* instead of the sound level *L*. For example, Fig 5B shows that the dissipation effect on *L* is 15 dB at a distance of 200 lattice spacings, while Fig 6 shows that the effect on Δ*L* is only 1 dB.

As explained before, there are two ways to reduce the dissipation effect in the LBM system:

1. reducing the lattice spacing Δ*x*, i.e. increasing the number of lattice points,
2. reducing the kinematic viscosity *ν*.

This section illustrates how these two approaches affect the sound level and the excess sound level.

**Fig 12:**
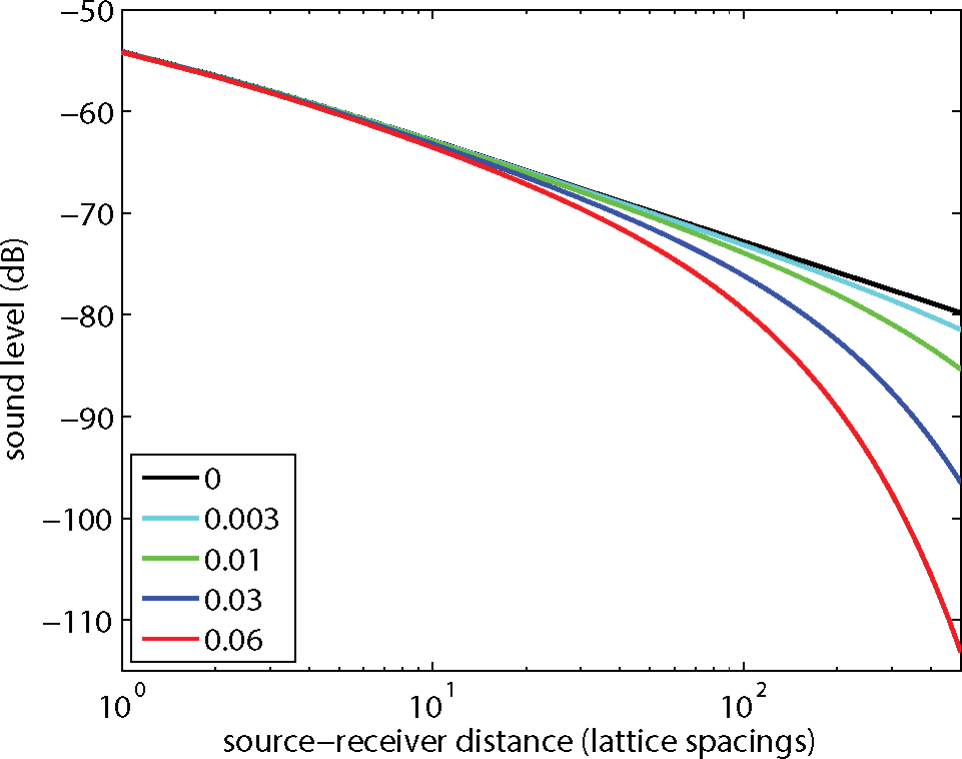
Free-field sound level as a function of source-receiver distance, for five values of the kinematic viscosity.

First the free-field sound level is considered. Fig 12 shows the sound level as a function of source-receiver distance, for five values of the kinematic viscosity. The curves were calculated with Eq (4), but LBM results agree well with the analytic solutions, except below a distance where near-field effects occur, as shown in Fig 4. This distance increases with decreasing viscosity: from about 3 lattice spacings for *ν* = 0.06 (see Fig 4) to about 20 lattice spacings for *ν* = 0.01, and about 80 lattice spacings for *ν* = 0.003. The LBM simulations were still stable for *ν* = 0.003, but for *ν* = 0.001 instabilities occurred.

**Fig 13:**
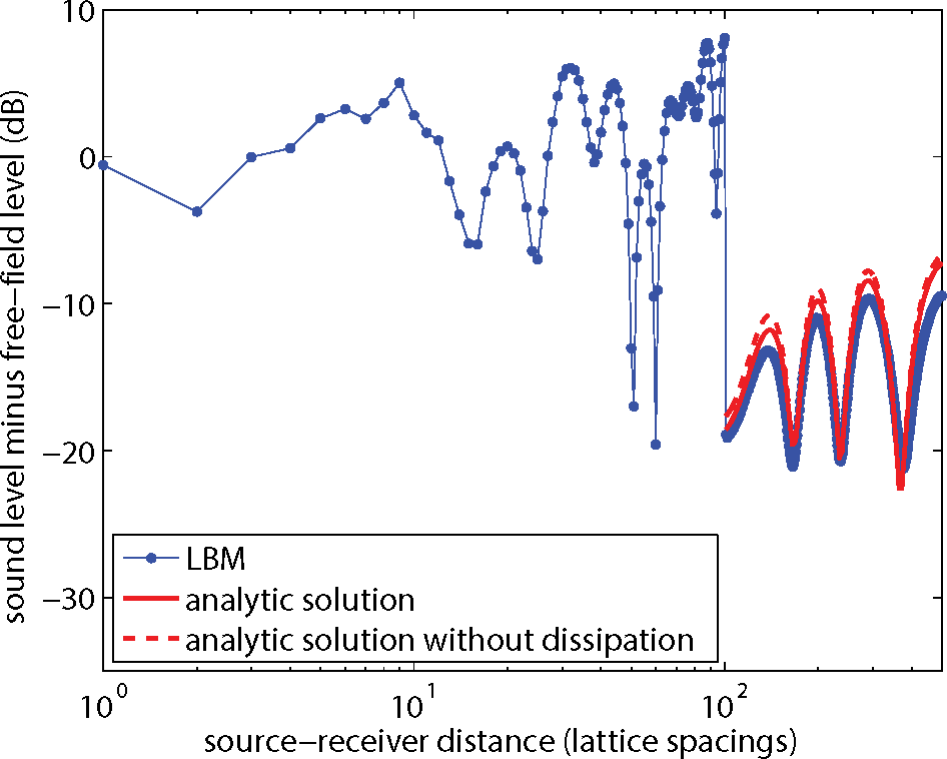
As Fig 11, but now for kinematic viscosity *ν* = 0.01.

**Fig 14:**
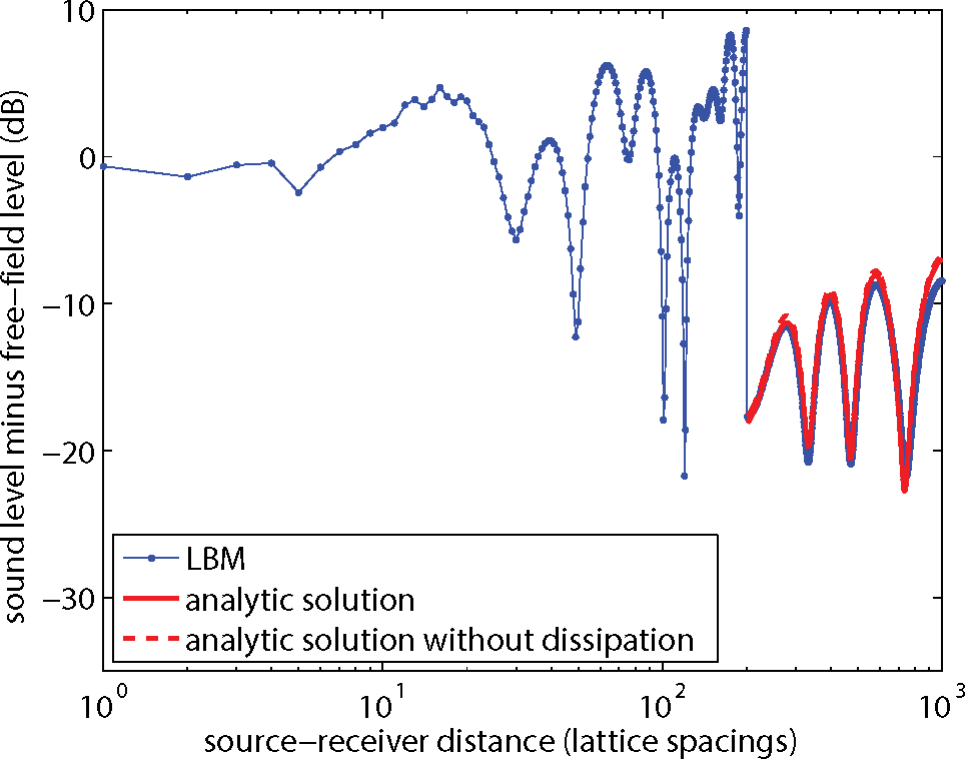
As Fig 13, but now for a system with halved lattice spacing.

Next the excess sound level Δ*L* for the system with the noise barrier is considered. The results shown in Fig 11 were for kinematic viscosity *ν* = 0.06 and lattice size 2000x1000. Fig 13 is similar to Fig 11, but now for kinematic viscosity *ν* = 0.01. The agreement between LBM results and the analytic solution is better than in Fig 11. The effect of dissipation is also smaller than in Fig 11. Fig 14 is similar to Fig 13, with *ν* = 0.01, but now the lattice spacing was halved, or equivalently a 4000x2000 lattice was used instead of a 2000x1000 lattice, and harmonic period *T* and lattice coordinates of source, receivers, and barrier were multiplied by a factor of 2. The differences between LBM results and the analytic solutions in Fig 14 are smaller than in Fig 13. The effect of dissipation is negligible in this case.

**Fig 15:**
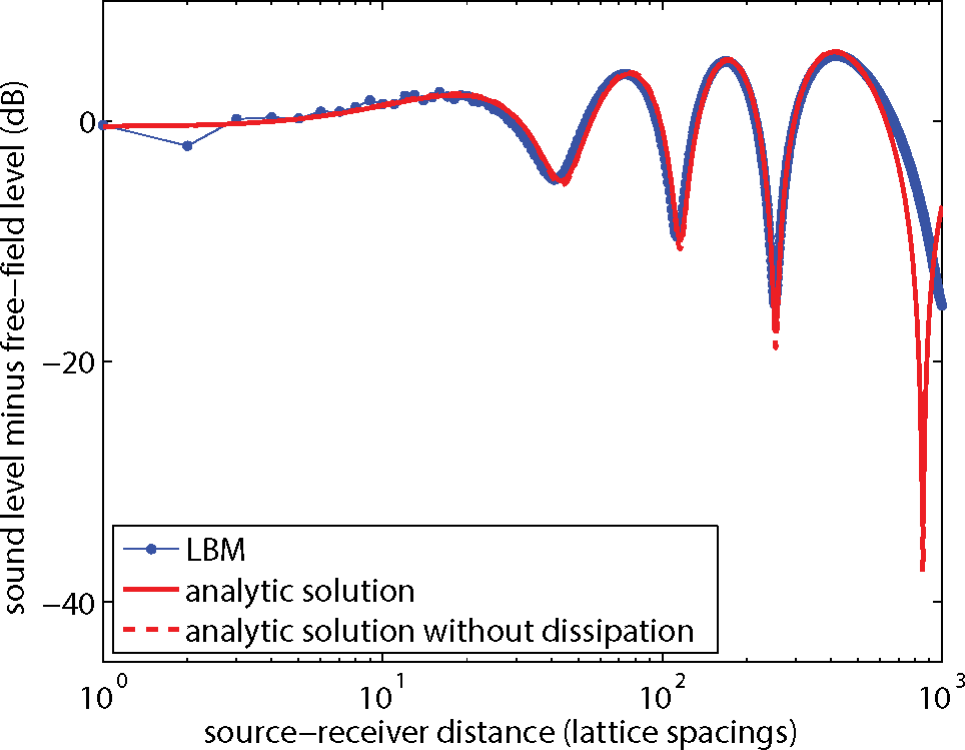
As Fig 6, but now for now for a system with *ν* = 0.01 and halved lattice spacing.

Fig 15 shows similar results for the ground effect. The figure is similar to Fig 6, but now for *ν* = 0.01 instead of *ν* = 0.06 and a 4000x2000 lattice instead of a 2000x1000 lattice. The agreement between LBM results and the analytic solution in Fig 15 is better than in Fig 6. The two analytic solutions in Fig 15 coincide, so the effect of dissipation is negligible in this case.

### Effect of Fluid Flow

The LBM can be used for calculating wind flow and sound propagation in a single simulation. This is an important advantage of the LBM for sound propagation studies. Wind effects on atmospheric sound propagation are large, in particular in situations with noise barriers [5]. An example of application of the LBM to fluid flow past an obstacle can be found in Ref. [33]. The LBM may be extended to include temperature gradients [34], which also affect sound propagation.

**Fig 16:**
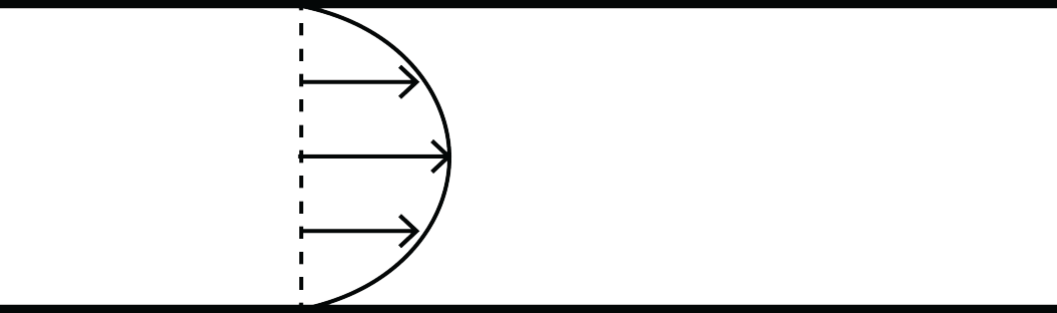
Schematic illustration of a Poiseuille flow profile.

To explore the performance of the LBM for sound propagation in an atmosphere with wind, a calculation was performed for a system with a sound source in a Poiseuille flow profile. Poiseuille flow occurs when fluid is forced through a pipe or between two parallel plates, while the fluid velocities at the walls are zero. The flow profile is parabolic, and is illustrated schematically in Fig 16.

An LBM calculation of Poiseuille flow was performed by including an external body force in the algorithm, as described in Ref. [3]. The external force is taken into account by modifying the velocity entering in the expression for the equilibrium particle distribution function. In this case there is a horizontal force to the right, so only the horizontal velocity component *u* is modified (*u* is replaced by *u*+*τg*, with force *g* = 1 ^**.**^ 10^-6^). At the solid walls first-order bounceback conditions were applied.

**Fig 17:**
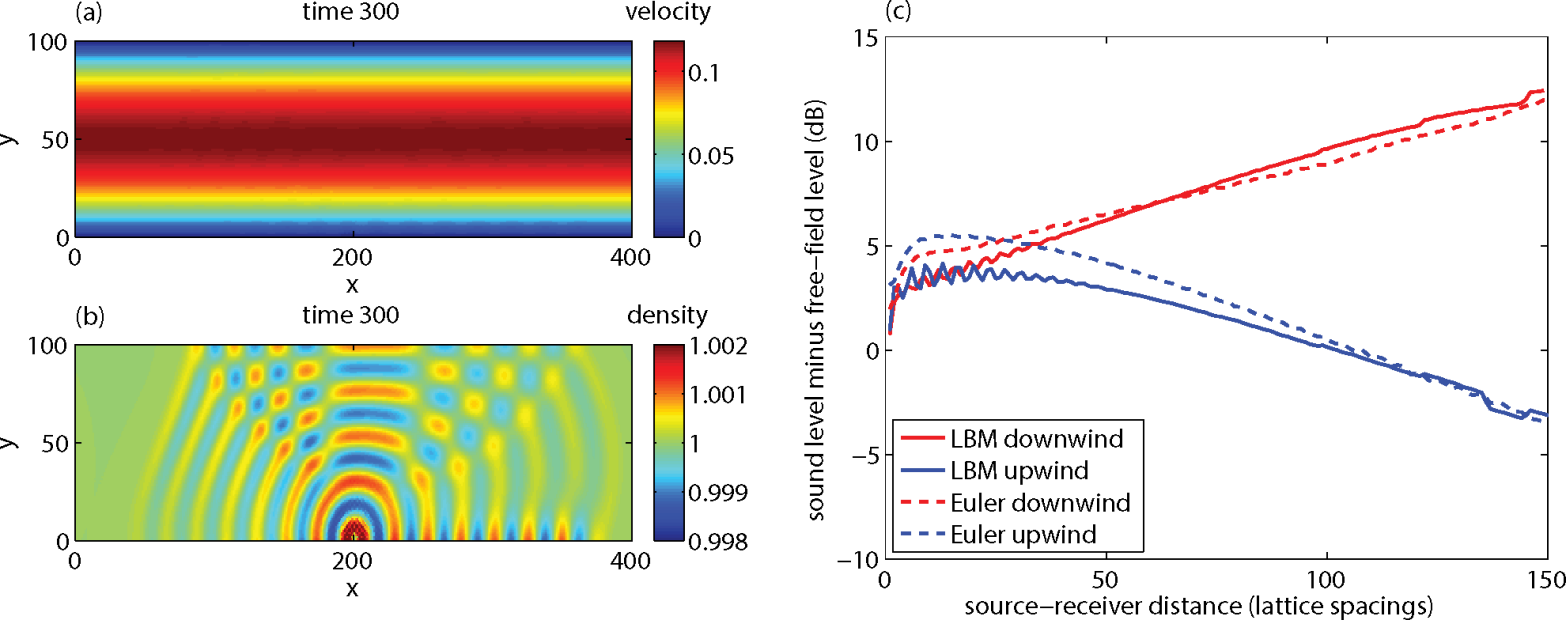
LBM results for a system with a sound source in a Poiseuille flow profile. Figure (a) shows the horizontal velocity component. Figure (b) shows the density field. Figure (c) shows the excess sound level Δ*L* at receivers near the lower wall (at the same height as the source, i.e. two lattice spacings above the lower wall).

An LBM lattice of 400x100 was used with *ν* = 0.01. First 2 ^**.**^ 10^6^ time steps were performed to reach a stationary Poiseuille flow profile. The velocity field is shown in Fig 17A. The maximum velocity occurs at *y* = 50, and the LBM value agrees well with the value of 0.1225 according to the analytic Poiseuille profile *u*(*y*) = 0.5⋅10^−4^[(*H* / 2)^2^ − (*y* − *H* / 2)^2^], with system height *H* = 99.

Next the sound source was switched on, with the same parameters as before (*B* = 0.01, *T* = 40). The source was located at *x* = 200, at a height of two lattice spacings above the lower wall. Fig 17B shows the density field after 300 time steps. The acoustic contribution to the density field is clearly visible as a deviation from the equilibrium density of unity. The acoustic contribution to the velocity field in Fig 17A is invisible, since it is small compared with the Poiseuille flow profile.

The focus is on receivers near the lower wall, at the same height as the source, i.e. two lattice spacings above the lower wall. In this way, the system is similar to a system with a source and a receiver near the ground in an atmosphere with wind. It can be seen in Fig 17B that the reflection from the top wall has not yet reached the receivers at time 300.

**Fig 18:**
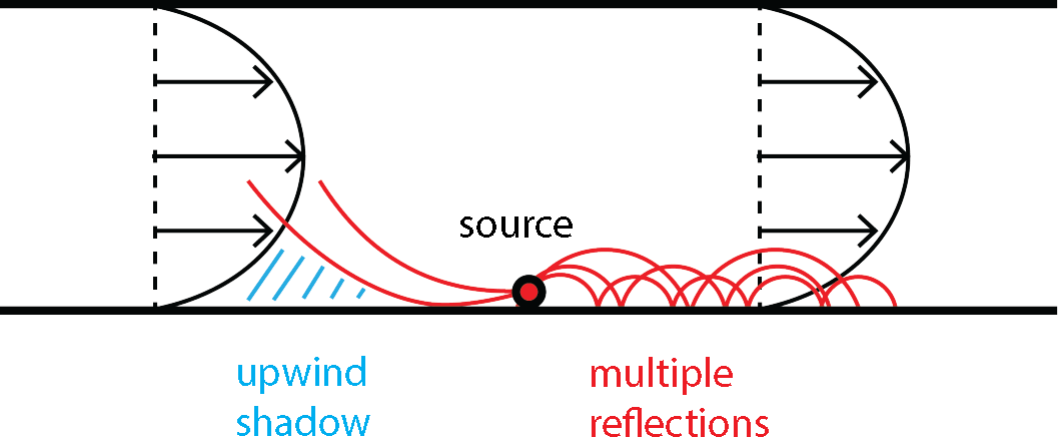
Schematic illustration of upward and downward refraction of sound waves. Red lines represent curved sound rays.

Fig 17B shows further that sound amplitudes at receivers on the downwind side of the source (to the right of the source) are higher than sound amplitudes at receivers on the upwind side of the source (to the left of the source). This is explained by Fig 18. On the downwind side, downward refraction of sound waves occurs, and multiple ground reflections lead to high amplitudes. On the upwind side, upward refraction occurs, and an acoustic shadow region with low sound levels occurs. For more information, see Ref. [6].

Fig 17C shows that the sound level difference between the downwind side and the upwind side is about 15 dB, at a distance of 150 lattice spacings from the source. Also included in Fig 17C are results of an Euler model, i.e. a numerical model for atmospheric sound propagation based on the linearized Euler equations [7–9]. The LBM results agree well with the results of the Euler model.

## Conclusions

This article has presented a practical approach for simulating sound propagation in the atmosphere with the lattice Boltzmann method (LBM) for fluid flow. LBM results were compared with solutions of the equations of acoustics, for various benchmark cases for outdoor sound propagation. The comparisons made it clear that the dissipation of sound waves in an LBM simulation is larger than the real dissipation of sound waves in air.

Consequently, *direct* application of the LBM in acoustics is restricted to cases with small propagation distances and low sound frequencies (i.e. cases where dissipation effects are negligible). Therefore, an *indirect* approximate approach was proposed: the LBM is used for assessing the *excess* sound level, i.e. the difference between the sound level and the free-field level. The effect of dissipation on the excess sound level is much smaller than the effect on the sound level. Consequently, the LBM can be used to estimate the excess sound level for a non-dissipative atmosphere, which is a useful quantity in atmospheric acoustics. With the indirect approach, the LBM can be used for a wider range of cases than with the direct approach. Future work should be aimed at further specification of the range of application of the LBM in acoustics.

The effect of dissipation on the excess sound level in an LBM simulation can be reduced in two ways:

1. by reducing the lattice spacing,
2. by reducing the kinematic viscosity.

For the cases considered in this article, it was shown that by choosing a small lattice spacing and a small kinematic viscosity

- the effect of dissipation on the excess sound level could be made negligibly small,
- agreement was obtained between LBM results for the excess sound level and analytic solutions.

Reduction of the kinematic viscosity is limited since the LBM is unstable for small values of the kinematic viscosity. Reduction of the lattice spacing is equivalent to choosing a larger number of lattice points. Larger LBM lattices correspond to larger calculation times. This underlines the importance of developing efficient LBM algorithms for parallel platforms to reduce calculation times.
